# Recent Advances in Photodynamic Imaging and Therapy in Hepatobiliary Malignancies: Clinical and Experimental Aspects

**DOI:** 10.3390/curroncol28050345

**Published:** 2021-10-11

**Authors:** Atsushi Nanashima, Masahide Hiyoshi, Naoya Imamura, Koichi Yano, Takeomi Hamada, Kengo Kai

**Affiliations:** Division of Hepato-Biliary-Pancreas Surgery, Department of Surgery, University of Miyazaki Faculty of Medicine, 5200 Kihara, Kiyotake, Miyazaki 889-1692, Japan; mhiyoshi@med.miyazaki-u.ac.jp (M.H.); naoya_imamura@med.miyazaki-u.ac.jp (N.I.); k01yano@med.miyazaki-u.ac.jp (K.Y.); takeomi_hamada@med.miyazaki-u.ac.jp (T.H.); kengo_kai@med.miyazaki-u.ac.jp (K.K.)

**Keywords:** photodynamic reaction, photodynamic eye imaging, photodynamic therapy, hepatobiliary malignancies

## Abstract

The therapeutic and diagnostic modalities of light are well known, and derivative photodynamic reactions with photosensitizers (PSs), specific wavelengths of light exposure and the existence of tissue oxygen have been developed since the 20th century. Photodynamic therapy (PDT) is an effective local treatment for cancer-specific laser ablation in malignancies of some organs, including the bile duct. Although curability for extrahepatic cholangiocarcinoma is expected with surgery alone, patients with unresectable or remnant biliary cancer need other effective palliative therapies, including PDT. The effectiveness of PDT for cholangiocarcinoma has been reported experimentally or clinically, but it is not the standard option now due to problems with accompanied photosensitivity, limited access routes of irradiation, tumor hypoxia, etc. Novel derivative treatments such as photoimmunotherapy have not been applied in the field hepatobiliary system. Photodynamic diagnosis (PDD) has been more widely applied in the clinical diagnoses of liver malignancies or liver vascularization. At present, 5-aminolevulinic acid (ALA) and indocyanine green (ICG) dyes are mainly used as PSs in PDD, and ICG has been applied for detecting liver malignancies or vascularization. However, no ideal tools for combining both PDD and PDT for solid tumors, including hepatobiliary malignancies, have been clinically developed. To proceed with experimental and clinical trials, it is necessary to clarify the effective photosensitive drugs that are feasible for photochemical diagnosis and local treatment.

## 1. Introduction

### 1.1. Photomedicine and Photodynamic Reaction

Photomedicine is a well-known therapy that utilizes various reactions with light irradiation for diseases including oncological malignancies, and it has been developed in the fields of dermatology, surgery, radiology or optical diagnostics, ophthalmology, infectology and oncology [1,2,3,4,5,6,7,8]. In photomedicine, photodynamic diagnosis (PDD) and therapy (PDT) involve the unique biological processes of photodynamic reactions with the delivery of suitable photosensitizers (PSs), which are transported via the vasculature after administration, such as vascular injection and oral intake [4,8,9,10]. Selective intracellular excitation and activation of PSs accumulate in target cells, including cancer cells, surrounding feeding vessels or immune cells, and are accompanied by direct tissue injury from light energy sources [11,12]. Although PSs exert cytotoxic or at least cytostatic effects due to sunlight or increased luces of light without specific irradiation and activation, after irradiation with a suitably specific wavelength of light, PS is detectable by fluorescence and reactive to oxygen species [13,14]. These are applied in diagnosis as well as in therapy [4]. Immune reactions are also induced, which may influence cellular immunity [4,15]. At increased lethal doses of PS, cells die by apoptosis, necrosis or autophagic cell death. Photodynamic therapy-induced apoptosis is conducted via the mitochondrial or receptor-mediated pathway [4,6,7,16,17]. In the field of hepatobiliary malignancies, the theme of this special issue in this journal, PDD and PDT appear to have advanced in the 21st century, which is supposed to be a dawn of “photon and photomedicine”. Dolmans or dos Santos et al. summarized the basis and clinical applications in 2003 [4,8]. At that time, PDT had only been tested in the clinic in oncology to treat various cancers, such as head and neck, brain, lung, pancreas, intraperitoneal cavity, breast, prostate and skin cancers, but had not been fully applied in hepatobiliary diseases at the end of the 20th century. In PS, talaporfin alone has been shown to accumulate well in the liver [8].

In the clinical background situation, the adequate treatment of cholangiocarcinoma in case of unresectable cases with multiple severe biliary stenosis, or the residual cancer treatment at the bile duct stump or bilio-intestinal anastomosis has not been established, while for liver malignancies, the efficient locoregional treatment for multiple lesion or extrahepatic lesion is required because these can be detected by the recent fluorescent diagnosis. By this point of view, we describe this review article.

### 1.2. Mechanism of Photodynamic Reaction and PS in Digestive Cancers

PDT involves two nontoxic components that combine cellular and tissue effects via an oxygen-dependent reaction and require two components: (1) PS: a photosensitive substance localized in specific cells or tissues and (2) irradiation of light of a specific wavelength activating the accumulated PS. PS in the ground (stable) state transfers light energy to an excited state, and tissue molecular oxygen transfers reactive free radicals or singlet oxygen [11,12,13,14]. The biological responses to PS are activated only in the parts of tissues or organs where they accumulate and upon which specific light exposure is focused. Another photochemical reaction causes direct injury to DNA or tissues [8,18]. PDT is thought to be involved in chemotherapy or brachytherapy for cancers, including biliary malignancies [19]. The most extensively applied PSs are porphyrin compounds. Most PDT occurs in an oxygen-dependent manner, and thus PS typically does not occur in anoxic tissue. The degree of photodamage and cytotoxicity depends on the type of PS, its extracellular and intracellular localization, the administered light energy dose and time, the oxygen availability, etc. A combination of components, such as direct cellular killing, vascular injury and immune responses, is required for long-term tumor control.

## 2. PDT

### 2.1. Effect on Tumors

Historically, hematoporphyrin accompanied by light exposure first exhibited cell toxicity in vitro in an animal cancer model and in cancer patients in the early 1990s. An interval between PS administration and light exposure was necessary until the maximum difference in PS concentration between target cancer tissues and surrounding normal cells was reached to achieve cancer toxicity and preserve normal tissues; this takes 24–72 h for porfimer sodium activated with an eximer dye laser (Photofrin; Wyeth Pharmaceuticals, Collegeville, PA, USA, and Wyeth, Tokyo, Japan) and 4–6 h for talaporfin sodium activated by a semiconductor laser (NPe6; Laserphyrin^®^ 100 mg for injection; Meiji Seika Pharma., Co. Ltd., Tokyo, Japan) [20]. As PS causes long-lasting cutaneous photosensitivity, patients who have been managed with PS must undergo shading from strong white light or sunlight for various periods, up to weeks. Photosensitivity of skin has been a problematic issue in patients undergoing PDT. After Dougherty’s clinical success for cancer therapy in 1983, PDT was attempted in esophageal squamous cell carcinoma, gastric or colorectal adenocarcinoma, cholangiocarcinoma and pancreatic adenocarcinoma [20,21,22]. Since the middle of the 1990s, successful treatment of skin disorders with 5-ALA has also been used for photodetection, photodiagnosis (described later) or PDT in malignant lesions [23].

### 2.2. PDT for Primary Liver Malignancies

The cytocidal effect of PDT on hepatocellular carcinoma (HCC) has been examined in vitro and in vivo in original English articles since 1997 [24,25,26,27,28,29,30,31,32,33]. In the clinically accepted PS worldwide, PDT using 5-ALA showed cytocidal molecular responses in an HCC cell line according to Abo’s report [24]. Recently, experimental PDT for HCC using PS under experimental compounds has been mainly reported by Chinese investigators [27,28,31,33]. However, the clinical effectiveness of PDT for HCC has not been clarified, although endoscopic laser irradiation of PDT for biliary obstruction by HCC might be attempted with multidisciplinary treatments [34,35]. Because the usefulness of near-infrared fluorescence imaging using indocyanine green (ICG) or 5-ALA (PDD, described above) was recently noted, the possibility of PDT for accumulated fluorescein substances in HCC in experimental HCC models would be expected [36,37,38,39]. Recently, nanoparticles composed of PS-based PDT showed cytocidal effects in HCC cells in the experimental setting [38,40,41]. PDT also causes microvascular damage in the tumor or peritumorous region, which leads to severe tissue hypoxia [42]. In the case of HCC or other hypervascular liver malignancies, the effectiveness of the clinical application of PDT under sufficient oxygen concentrations is promising for clinical development in the near future. In our experiment using a novel highly water-soluble porphyrin (phosphorus tetraphenyl-porphyrin), the cytocidal effect of PDT using the low energy light-emitted diode (LED) was highly observed in various malignant cells but not in HepG2 human hepatoma cells, which are tolerant to porphyrin compound-based PDT [43,44]. However, by modifying glucose-conjugated PS, the tumor viability of HepG2 cells dramatically decreased; therefore, HepG2 cells may also be integrated with the development of PS (not published yet), as noted in a recent report [45]. Preclinical trial of PDT using ICG for primary liver cancer seemed to be scheduled by the animal model [37]. With respect to intrahepatic cholangiocellular carcinoma as a mass-forming type, except for perihilar cholangiocarcinoma, no clinical reports have been observed. In our preliminary (preclinical research) report in 2004, one case of recurrent intrahepatic cholangiocarcinoma at the transected stump received an endoscopy applied PDT with a porfimer sodium and eximer dye laser, and the obstructive jaundice was relieved for two months [46]. However, regrowth of remnant tumors was rapid, and effectiveness was not shown. For intrahepatic peripheral lesions, irradiation of PDT would be mainly performed via transhepatic surface using laparoscopy or open laparotomy.

### 2.3. Extrahepatic Cholangiocarcinoma

**Figure 1:**
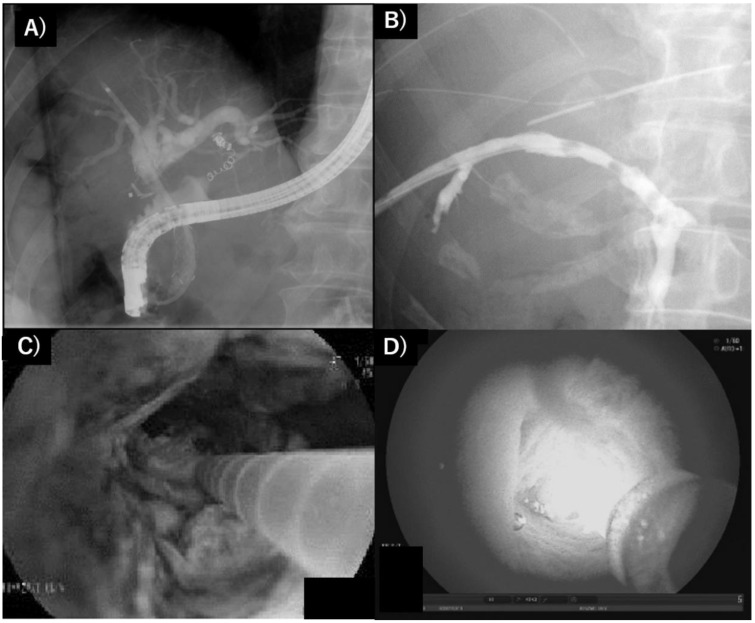
Endoscopic approaches for laser irradiation for PDT. (**A**) The transhepatic biliary drainage route; (**B**) intraductal placement image of laser fiber adjacent to tumor; (**C**) laser irradiation using laser fiber generated by the eximer dye laser or semi-conductor apparatus via endoscopy; (**D**) Cholangioscopic findings of laser irradiation at the biliary stricture due to invading cholangiocarcinoma.

**Figure 2:**
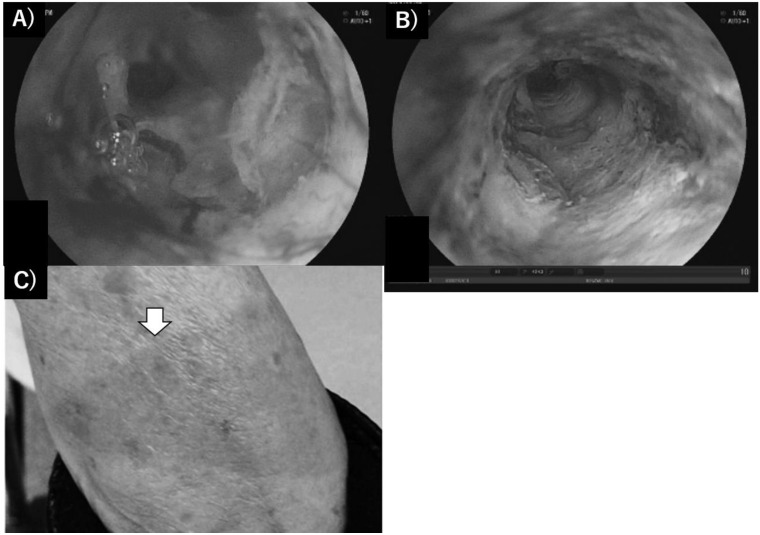
(**A**) Cholangioscopy findings showed encircled cancer stenosis; (**B**) stenosis was released by cancer shrinkage with a white necrotic plaque and reddish tissues after PDT; (**C**) mild photo-reaction of the forearm at day 7 after PDT.

In the late 1990s, basic research and preclinical trials for extrahepatic, periductal invasive-type cholangiocarcinoma or large bile duct-origin adenocarcinoma were performed worldwide. In the basic study, certain reports elucidated that cholangiocellular carcinoma was sensitive to cytocidal effects by PDT using porphyrin chemical compounds, although cholangiocarcinoma is not a relatively hypervascular malignancy [22,47,48,49,50,51,52,53]. Pahernik reported that the accumulation of porfimer sodium in the bile wall was significantly increased in comparison with that in surrounding peripancreatic organs, such as the gall bladder, pancreas or duodenum, in humans, and the maximum difference duct in the accumulated tissue concentration of PS between 24 and 48 h after administration was over twice that between normal tissue and cancer [48]. Talaporphin sodium also accumulated, and the concentration of fluid was increased in bile juice [54]. Thus, cholangiocarcinoma was considered to be a good target of PDT if light exposure was possible in the late 1990s. In the clinical setting, PDT has been attempted for biliary stricture of cholangiocarcinoma via a transhepatic or jejunal biliary approach since the 1990s [55,56]. Rumalla first reported successful results of PDT for cholangiocarcinoma using an endoscopic transpapillary approach with a monorail procedure [57]. Consecutive clinical reports from Germany or other investigators in Europe with respect to the usefulness of PDT to amend biliary obstruction and impaired patient prognosis by cholangiocarcinoma have revealed great impacts worldwide, which were clarified by prospective or randomized multi-institutional trials, including small cohorts, between 2003 and 2006 [57,58,59,60,61,62]. The effectiveness of PDT for longer patient prognosis by developing novel PSs such as temoporfin (Foscan^®^, Biolitec Pharma Ltd., Dublin, UK) or combined systemic anticancer chemotherapy has been reported to date [63,64,65,66,67]. However, UK reports by Pereira revealed contradictory results that the delayed induction of effective chemotherapy due to PDT management for a long period led to the lower overall survival period in patients with cholangiocarcinoma in a randomized control trial [68]. Although the first and second editions of the evidence-based Japanese guidelines also refereed the possibility of PDT for unresectable cholangiocarcinoma [69], the option of PDT has not been described in the recent third guideline. Several investigators attempted PDT for cholangiocarcinoma in the adjuvant setting. Berr et al. showed the histological evaluation of cancer necrosis by neoadjuvant PDT; however, subsequent reports clarifying the necrotic effects of cancer tissue have no longer been reported thus far [70,71]. For lesions of the large biliary system, endoscopic irradiation is adequate and, therefore, mass-forming type cholangiocarcinoma would be inadequate via endoscopic route. While, in the novel experimental setting, certain promising candidates of PS for PDT in the cholangiocarcinoma have been reported by any ingenuity as chlorin e6 derivative Chlorin A, zinc phthalocyanine (ZnPc), anti-cancer drug loaded DSPE-PEG-PheoA liposomefor the recent a few years [72,73,74]. Figure 1 and Figure 2 showed our experienced cases of PDT in patients with cholangiocarcinoma.

### 2.4. Limitation and Disadvantages of PDT in Hepatobiliary Malignancies

A large cohort of PDTs for hepatobiliary malignancies has not yet been reported. The several reasons were considered as follows. (1) The detailed data of PS delivery to or accumulation in both normal and targeted cancer tissue were uncertain because the cholangiocarcinoma or bile duct samples were too small in unresectable patients. (2) The accompanying photosensitivity by PS often leads to severe adverse effects of dermatitis. Therefore, complicated management, including longer periods under sunlight shielding, is required to avoid photosensitivity [75]. (3) Simultaneous combination therapy with systemic anticancer drugs or other treatments has not been fully established. (4) The access route of light (laser) exposure has not been established, particularly the limited space of biliary tracts [76]. (5) The permeability of light exposure that leads to sufficient PDT activity is still unknown in limited space and maintaining the stability of light exposure strength leading to sufficient PDT activity is difficult. In the case of biliary stricture, a single light exposure might not be enough. Furthermore, the efficacy or safety of PDT under the replacement of biliary stenting (tube or metal) is still uncertain [68]. (6) Targeted tumor locations cannot be precisely detected because the excited light wavelength is inadequate for PDD at present. In the case of cholangiocarcinoma, PDD has not been established thus far. (7) Particularly in Japan, the cost of PS and the apparatus is still high; therefore, the health economic balance has not improved. How is the cost in European countries? In the case of liver cancer, although light exposure may be feasible for the liver surface via laparoscopy or laparotomy, the permeability of light exposure would become a similar limitation in the deeper part or in deeply located tumors, as well as in photodynamic eye imaging described later.

## 3. PDD

### 3.1. PDD during PDT

Diagnostic tools using photoreaction for autofluorescence diagnosis or fluorescein-conjugated immunostaining in malignancy are different from photodynamic methods; therefore, the former is not described in this chapter. The photodynamic effect can be used to detect tumors, and Dolmans detected the localization of PS to the vascular endothelium of breast cancer in vivo in 2002 [77]. Hematoporphyrin, porphyrins, hematoporphyrin derivatives (HPDs) and ALA derivatives were tested for use in tumor detection [78]. Clinically, PDD for cancer detection has been attempted since the 1950s, and these agents showed contrast between squamous cell carcinoma and normal tissue between the 1960s and 1990s [4], but there were no remarkable reports in the case of cholangiocarcinoma or HCC up to the 2000s. In our series, photodetection by optical endoscopy with a commercial filter lens for a specific wavelength was attempted during PDT in patients with cholangiocarcinoma or bronchial cancer; however, clear contrast of the PS accumulated area could not be confirmed (data not indicated) [46]. In animal experiments with a subcutaneously transplanted bile duct cancer cell line (NOZ), PDT using glucose-conjugated chlorin showed an increase in fluorescence intensity at the tumor; however, clear contrast with normal mouse skin could not be detected [52].

### 3.2. Recent PDD Using ALA and ICG-Based NIR Fluorescence Imaging

**Figure 3:**
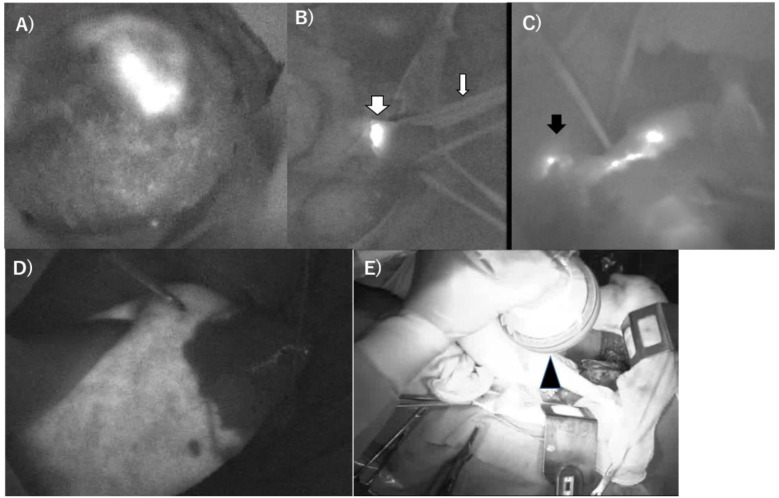
Representative image of the ICG-photodynamic eye image during operation in a hepatocellular carcinoma patient, which showed (**A**) fluorescence of the main HCC and (**B**) portal vein tumor thrombus in the right portal vein (thick white arrow). The thin arrow showed a taped each portal vein and trunk. (**C**) A case of hepatic venous tumor thrombus (black arrow). (**D**) Identification of anatomical demarcation border. (**E**) Observation by the ICG-PDE camera at operative field (Black arrowhead, Hamamatsu Photonics, Hamamatsu, Japan).

PDD using 5-ALA and near-infrared (NIR) fluorescence imaging with ICG has been dramatically applied worldwide in the clinical setting to detect vascular or lymphatic flow or to detect hypervascular cancer in the 2010s [79,80,81]. First, 5-ALA is a precursor of protoporphyrin IX (PpIX), which is a photosensitive compound, PS, and accumulates specifically in cancer cells. By irradiation with 375–445-nm blue light exposure, the excited intracellular PpIX emits 600–740 nm red light. 5-ALA is metabolized to PpIX in mitochondria. In cancer cells, the biosynthesis of PpIX is promoted, but the metabolism of PpIX is conversely suppressed compared to normal cells [79]. As a result, this marked accumulation in cancer cells can be visualized using the appropriate detectors. PDD using 5-ALA is possible for liver cancer cell lines, and in addition, the effect of PDT using 5-ALA is still controversial in vitro [24,82,83]. A clinical trial of 5-ALA PDD for patients with hepatocellular carcinoma and metastatic liver cancer during hepatectomy was attempted by Inoue, and they concluded that residual tumors at the cut surface of the remnant liver were improved by PDD with 5-ALA [84]. For cholangiocarcinoma, 5-ALA PDD is feasible in a cholangiocarcinoma mouse model [85]. At present, PDD with 5-ALA is an experimentally promising state for hepatobiliary malignancies. Fluorescence imaging with ICG for malignant hepatic tumors has been rapidly approved since Ishizawa’s report in 2009 [86]. ICG is a pigment dye that has been widely used clinically for liver function examinations since the late 1950s, [79,87] in which the molecular energy state increases to emit a higher energy radiation of NIR and accumulated region excited light exposure can be visualized by exclusive detectors [79,88]. ICG binds to plasma proteins and is removed from circulation exclusively by the liver to the bile juice; therefore, it might be trapped in the hypervascular area of the hepatobiliary system as malignant tumors. The absorption and fluorescence spectrum of ICG is in the near infrared region. ICG emits fluorescence at a specific wavelength of light in the near-infrared spectrum (approximately 835 nm) when excited by light with a wavelength of 760 nm. ICG-guided surgery has been recently developed, and real-time cancer or blood flow visualization in the hepatobiliary region can be achieved at present [89,90,91]. In addition to HCC, the hypervascular area of intrahepatic cholangiocarcinoma, hepatoblastoma, and [89] the area surrounding metastatic colorectal cancer have been well examined [92]. Although NIR light cannot be seen with the naked eye, the distribution of subtle fluorescent materials in tissues can be precisely detected through dedicated CCD camera, which has been conventionally applied. Furthermore, the detection of intraabdominal metastatic lesions of HCC can also be applied [93,94,95]. ICG photodynamic eye imaging equivalent to PDD allows segmental visualization of hepatic blood flow, which has been useful for anatomical liver resections [79,90,91,96]. In 2021, the first worldwide consensus guideline for the fluorescence imaging using ICG in hepatobiliary Surgery has been eventually published [97]. Cyanine dye also has photosensitizing characteristics [98]. Experimental reports regarding PDT with ICG and its combination with other chemical compounds, such as nanoparticles, for liver cancer have recently increased [36,99,100]. In biliary cancers, Hishikawa et al. reported the usefulness of NIR fluorescence imaging and photodynamic therapy with ICG lactosomes in gallbladder cancer cell lines [101]. Furthermore, photoimmune therapy, chemotherapy, and thermal therapy have been experimentally developed in a liver cancer model. Thus, the clinical application of PDT using the novel PS is promising for hepatobiliary malignancy treatment in the future [102,103,104]. Figure 3 showed our experienced cases of PD imaging in HCC patients.

### 3.3. Limitation, Disadvantages of PDT in Hepatobiliary Malignancies

Similar to PDT, detection of tumor or vascularity in hepatobiliary lesion is limited due to light penetration in the parenchymal organs as liver or hepatoduodenal ligament because the deeper part less than approximately 10 mm cannot be visualized by the latest CCD camera from the surface thus far [89,93,94,95], although the intrahepatic transected boundary seems to be detected. PDD for malignancies is depended to tumor vascularity and, further, false positive surrounding lesion as liver cyst, cirrhotic nodule, hemangi-oma [96].

## 4. Problems and Debates

Issues to solve issues for the future application were summarizes in this part. PDD or PDT require the three components of PS accumulation, light exposure for excitation and cellular oxygen; therefore, all components are necessary to achieve PDT cytotoxicity in cancer cells clinically [4]. Endoscopy is necessary for the access route of light exposure. In hepatobiliary diseases, a specific wavelength light source is optimally placed as a laser fiber via cholangioscopy or laparoscopy. At present, a special and expensive light generator apparatus is required for each PS. Therefore, this modality is for local but not systemic photochemical diagnosis or treatment; thus, combined systemic chemotherapy is usually necessary. What is the real indication of these modalities in the era of multidisciplinary options for cancer treatment that have been developed in this malignancy? One is supposed to release the obstructive jaundice by malignant biliary strictures, and another is an additional treatment for remnant cancer cells at the edge of surgical resection, the transected plane of liver parenchyma or a stump of bile duct wall [20,46,105], because remnant cancer positivity is significantly associated with patient survival. To avoid the photosensitivity or photoinjury of normal tissue, the development of cancer-specific accumulation or photoreaction of PS is necessary. NIR-induced photoimmunotherapy applying monoclonal antibodies to cancer-specific antigens is promising [106]. Induction of systemic immune reaction by this modality is expected. However, in hypoxic tumor environments such as cholangiocarcinoma, it will be difficult to produce reactive oxygen species as singlet oxygen species by PDT in the center or deep part of the tumor [4,79]. Light penetration into the central or deeper part of cancer tissue may be attenuated in both PDT and PDD. Lasers have been applied for excitation of PS in PDT; however, light emitted diodes (LEDs) are also expected to be used because of their economically cheaper cost and the safety due to their low energy at direct exposure [107,108]. PDT can be achieved by recent reports, including our experimental results [20,43,44,46,52,105,109]. Eventually, concomitant procedures using both PDD and PDT are ideal; however, no successful report has been seen in the clinical setting. If the combination of two kinds of photosensitive chemical substances for tumor fluorescence detection and high cancer-cellular toxicity in each is developed, both PDD and PDT can be achieved. Although applying the existing PS for PDT is considered, we must develop novel PS discoveries in future photodynamic medicines, particularly for the treatment of hepatobiliary malignancies.

## 5. Conclusions

It has been over 100 years since the first PDT report, and PDT has been developed as an experimental clinical modality. In Eastern and Western countries, several PSs have been approved for clinical use for the treatment or diagnosis of malignancies, including hepatobiliary diseases. Overall, PDT is potentially promising, depending on the limited indications in comparison with the wide clinical development of PDD. However, there are many problems associated with the use of PDT in a large series. In the future, it is likely that PDT will continue to be used as a stand-alone modality or in combination with chemotherapy, surgery, radiotherapy or other new strategies. Achieving ideally combined PDT with PDD provides a new cancer treatment strategy, including in refractory hepatobiliary malignant diseases, in the future.
